# The levels of CD4+CD25+ regulatory T cells in patients with allergic rhinitis 

**DOI:** 10.5414/ALX01782E

**Published:** 2018-09-01

**Authors:** Y. Shaoqing, C. Yinjian, Y. Zhiqiang, Z. Ruxin, C. Na, G. Rongming

**Affiliations:** 1Department of Otolaryngology, Tongji Hospital, Tongji University, Shanghai,; 2Department of Laboratory Medicine, Jinan General Hospital of PLA, Shandong,; 3Department of Otolaryngology, Xuzhou 97th Hospital of PLA, Jiangsu,; 4Department of Otolaryngology, Huadong Hospital, Fudan University, Shanghai, China

**Keywords:** allergic rhinitis, CD4+CD25+ regulatory T cells, flow cytometry, serum IgE, Th lymphocyte

## Abstract

Background: The involvement of CD4^+^CD25^+^ regulatory T cells (CD4^+^CD25^+^ T_Regs_) in allergic diseases was reported previously. However, it remains unclear whether CD4^+^CD25^+^ T_Regs_ are involved in allergic rhinitis (AR). Methods: Fresh whole blood from 20 patients with AR and 16 healthy donors was used to investigate the frequency of CD4^+^CD25^+^ and CD4^+^CD25^hi^ Treg cells using flow cytometry. In addition, serum total IgE (IU/mL) levels were determined using enzyme-linked immunosorbent assays. Results: Patients with AR had fewer CD4^+^CD25^+^ Treg cells (2.80 ± 1.36% vs. 3.94 ± 0.97%, P < 0.01) and CD4^+^CD25^hi^ T_Regs_ (1.53 ± 0·62% vs. 2.00 ± 0.52%, P < 0.05) than control subjects. The number of CD4^+^CD25^+^ and CD4^+^CD25^hi^ T_Regs _was correlated negatively with total immunoglobulin E levels (r = –0.79, P < 0.01 and r = –0.61, P < 0.01, respectively). Conclusion: Deficient regulatory T cells might play a role in the development of AR.

**German version published in Allergologie, Vol. 39, No. 3/2016, p. 109-115**

## Introduction 

Allergic rhinitis (AR) is an example of a persistent inflammatory disease of the nasal mucosa. It is caused by the secretion of interleukin (IL)-4, IL-5, and IL-13 by CD4+ Th2 effector cells in response to harmless environmental antigens [1]. The T cells that infiltrate the nasal mucosa are predominantly Th2 in nature, and they release cytokines that promote immunoglobulin E (IgE) production by plasma cells. In turn, IgE production triggers the release of mediators, such as histamine and leukotrienes, which are responsible for arteriolar dilation, increased vascular permeability, itching, rhinorrhea, mucous secretion, and smooth muscle contraction[2]. Recently, regulatory T cells (T_Regs_) were identified as being essential for immune tolerance. In addition, effector Th lymphocytes and T_Regs _play important roles in AR [3]. 

Approximately 15% of human peripheral blood CD4 T cells can express CD25, which is the IL-2 growth factor receptor α-chain. However, the CD25^hi^ cells represent only 1 – 2% of the total CD4+ T cell population, while the CD25^low^ cells can represent up to 16% of CD4^+^ T cells [4]. Thus, CD4+CD25^hi^ T cells can mainly represent CD4^+^CD25^+^ Treg. CD4^+^CD25^+^ T_Regs _account for 5 – 10% of CD4^+^ T cells in healthy humans and play a critical role in preventing organ-specific autoimmunity and allograft rejection as well as maintaining self-tolerance by preventing the activation and proliferation of autoreactive T cells that have escaped thymic deletion [5, 6] The transition from the early activation stage to the differentiated Th2 state might be blocked in patients by CD4^+^CD25^+^ T_Regs_ specifically, which limits airway allergic inflammation and prevents the inappropriate Th2 responses to environmental allergens [7, 8]. Several independent lines of evidence suggest that the number or function of T_Regs _is impaired or altered in patients with allergies compared with healthy individuals [9, 10]. 

Some studies suggest that the number and function of CD4^+^CD25^+^ T_Regs _is not impaired in patients with allergies, whereas other reports indicate that a decreased number of CD4^+^CD25^+^ Treg cells might be related to allergic disease [11, 12, 13, 14]. However, the role of T_Regs _in the pathogenesis of allergic disease was not defined until recently, and data concerning the role of T_Regs _in the pathogenesis of AR are rare. 

The aim of the current study was to investigate the role of T_Regs _in the pathogenesis of AR by exploring the CD4^+^CD25^+^ Treg population during AR, and to determine whether the number of T_Regs _was associated with disease severity in patients with AR. 

## Materials and Methods 

### Study participants 

We selected 20 patients with persistent AR and 16 age-matched healthy control subjects without allergies. Table 1 shows the subjects’ clinical characteristics. AR was diagnosed if an IgE-mediated response, such as nasal itching, sneezing, watery rhinorrhoea and/or nasal stuffiness, was induced after allergen sensitization. Patients in the AR group had symptom scores of at least 6 as assessed by the Total 5 Symptom Score (T5SS, including rhinorrhea, sneezing, nasal congestion, and nasal and ocular pruritus) [15]. Serum total IgE (IU/mL) levels were determined by an enzyme-linked immunosorbent assay method. Subjects in the non-allergic group had no history of allergies, a negative skin-prick test using a panel of common aeroallergens (mixed grass pollen, tree pollen, weeds, house-dust mites, cat hair, dog hair, and mold), and were negative for IgE. Patients with other complications, such as asthma or sinusitis, were also excluded. The study was approved by the Institutional Review Board at the Tongji Hospital, Tongji University, Shanghai City, China. 

### Antibodies 

The following monoclonal antibodies against human cell-surface molecules were purchased from Becton-Dickinson Immunocytometry Systems, San Jose, CA, USA: fluorescein isothiocyanate-conjugated mouse anti-human CD4 (CD4-FITC), phycoerythrin-conjugated mouse anti-human CD25 (CD25-PE), FITC-conjugated mouse immunoglobin G1 isotype control (IgG1-FITC), and PE-conjugated mouse immunoglobin G1 isotype control (IgG1-PE). 

### Flow cytometric analysis 

All antibodies were used at concentrations that were determined to be optimal for staining in antibody titrations. Briefly, a sample of whole blood was incubated with 5 μL CD4-FITC and 5 μL CD25-PE or isotype control (IgG1-FITC and IgG1-PE) in the dark for 30 min at room temperature. They were then washed with phosphate-buffered saline twice, and fixed with 1% paraformaldehyde at room temperature. Then, two-color immunofluorescence analysis was performed using MPL FC 500 (Beckman Coulter, Brea, CA, USA). Data analysis was performed using CXP software. An appropriate gate was drawn around the lymphocyte population, as defined by forward scatter and side scatter characteristics. The gated cells were then analyzed for CD4 and CD25 expression, and CD25^+^ cells were distinguished from those with a distinct (> 7-fold) brighter fluorescence signal as CD25^hi^. 

### Statistical analysis 

Standard two-tailed t-tests or nonparametric tests for two independent samples were used for the statistical analyses. Data are presented as the means. Spearman correlations were used to analyze relationships between variants. p values of < 0.05 were considered to be significant. All analyses were performed with statistical software SPSS version 17.0 (SPSS Inc., Chicago, IL, USA). 

## Results 

### Characteristics and IgE values of study participants 

The clinical characteristics of the study cohort are shown in Table 1. The subjects in each group were matched for age and gender. Patients with persistent AR had significantly higher serum total IgE values than did healthy individuals (312.8 ± 165.8 IU/mL vs. 70.1 ± 14.6 IU/mL, p < 0.01). 

### Decreased numbers of CD4^+^CD25^+^ and CD4^+^CD25^hi^ Treg cells in AR patients compared with control subjects 

**Figure 1. Figure1:**
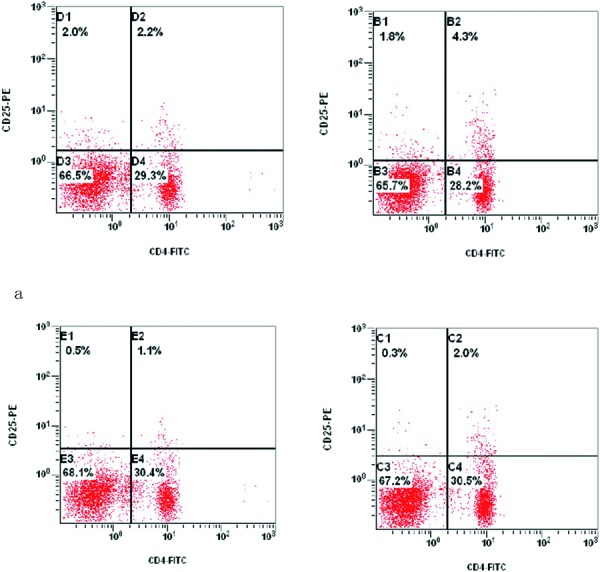
Flow cytometric analysis of CD4+CD25+ (a) and CD4+CD25hi (b) Tregs in AR patients (left) and healthy controls (right). Peripheral blood mononuclear cells (PBMCs) were isolated from fresh blood and stained with immunofluorescent antibodies against Treg surface markers (PE-conjugated anti-CD25 and FITC-conjugated anti-CD4 antibodies). Dots in the right upper quadrants each show an individual cell that stained positive for CD4 and CD25

**Figure 2. Figure2:**
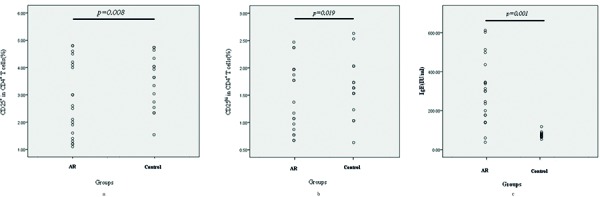
Comparison of the numbers of CD4+CD25+ (a) and CD4+CD25hi (b) Tregs and serum total IgE (c) levels between control subjects and AR patients. The number of CD4+CD25+ and CD4+CD25hi Tregs was decreased significantly, whereas serum total IgE levels were increased in AR patients.

As shown in Figures 1 and Figure 2, patients with AR had significantly decreased numbers of CD4^+^CD25^+^ T lymphocytes compared with control subjects (2.80 ± 1.36% vs. 3.94 ± 0.97%, p < 0.01) (Figure 2a). The numbers of CD4^+^CD25^hi^ T_Regs _were also decreased significantly in AR patients compared with control subjects (1.53 ± 0.62% vs. 2.00 ± 0.52%, p < 0.05) (Figure 2b). 

### Negative correlation between total serum IgE levels and the number of T_Regs_


**Figure 3. Figure3:**
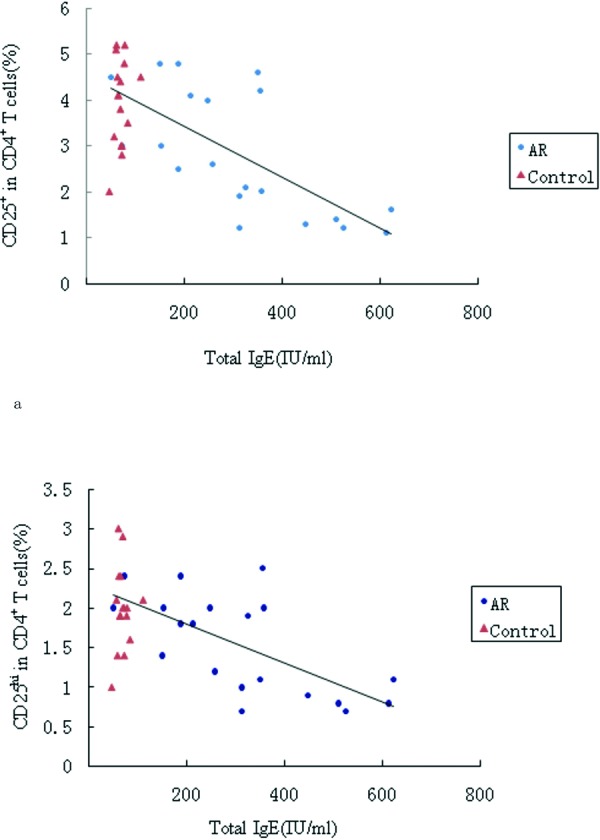
Correlation between the numbers of CD4+CD25+Tregs (a) and CD4+CD25hi Tregs (b) and serum total IgE levels. The numbers of CD4+CD25+ Treg cells and CD4+CD25hi Treg cells were both negatively correlated with total IgE levels (r = −0.66, p < 0.01 and r = −0.61, p < 0.01, respectively).

Serum total IgE levels were measured in all patients to investigate the association between AR and the number of T_Regs_. Serum total IgE levels were increased significantly in AR patients compared with control subjects (312.8 ± 165.8 vs. 70.1 ± 14.6, p < 0.01) (Figure 2c). The number of T_Regs_ and CD4^+^CD25^hi^ T_Regs _were negatively correlated with total serum IgE levels (r = −0.79, p < 0.01; r = −0.61, p < 0.01, respectively) (Figure 3a and b). 

## Discussion 

The activation of Th2 cells is thought to play an important role in allergic reactions by mediating IgE synthesis and eosinophilic inflammation via IL-4, IL-5, and IL-13 [16, 17]. Allergen-induced T cell proliferation can be detected in primary cultures of peripheral blood mononuclear cells (PBMCs) from individuals both with and without allergies. However, PBMCs from allergy patients produce increased levels of Th2-type cytokines including IL-4, IL-5, and IL-13 [18, 19]. However, it is unclear why subjects with allergies elicit a Th2-type T-cell response whereas other individuals do not. One hypothesis is that Treg-based mechanisms prevent the IgE responses to allergens in normal individuals, including the suppression of Th2 responses by regulatory T lymphocytes. 

T_Regs _control the development of autoimmune disease and transplant rejection, and also play a major role in regulating allergic reactions including AR and asthma. A distinct type of T_Regs _was identified in mammalian and humans, CD4^+^ T cells, which are characterized by the expression of the surface marker CD25 surface marker, and account for 5 – 10% of the normal CD4^+^ T cell population [5, 20]. Autoreactive T cells are also present in the peripheral blood of healthy individuals without any evidence of autoimmune disease, suggesting that some mechanisms regulate the autoreactive T cells to prevent autoimmune disease. CD4^+^CD25^+^ Treg cells play a critical role in the induction and maintenance of peripheral self-tolerance. Specifically, they prevent the activation and proliferation of the potentially autoreactive T cells that have escaped thymic deletion [21, 22, 23, 24]. CD4^+^CD25^+^ Treg cells play a key role in modulating Th2-mediated pulmonary inflammation by suppressing the development of the Th2 phenotype, which effectively promotes airway eosinophilia in-vivo[8]
The specific mechanisms by which CD4^+^CD25^+^ Treg cells function and their specific characteristics are still being investigated. 

The current study suggested that a lower frequency of CD4+CD25+ Treg cells is associated with the pathogenesis of AR. In addition, the number of T_Regs _was correlated with the inflammation status, as determined by measuring serum total IgE levels, in subjects with AR, but not in healthy subjects. These observations are consistent with previous observations in patients with allergic asthma. Xue and Shi investigated the number and functional role of CD4^+^ CD25^+^ T_Regs _in the PBMCs of patients with allergic asthma [25, 26]. They used flow cytometry to reveal a decrease in the CD4^+^CD25^+^ Treg ratio of CD4+ T cells and function in the PBMCs of patients with asthma. The authors concluded that CD4^+^CD25^+^ T_Regs _are critical for maintaining self-tolerance, and are associated with moderate to severe asthma. Consistent with this, Ling et al. [27] reported that the number of allergen-specific Th2 cells in allergic patients was enhanced significantly by the depletion of CD4^+^CD25^+^ Treg cells. Therefore, CD4^+^CD25^+^ T_Regs _play a crucial role in preventing inappropriate Th2 responses in allergic diseases. 

However, other studies contradict the above findings. For example, Han et al. showed that the number and function of CD4^+^CD25^+^ T_Regs _from patients with allergies was not impaired [28]. In addition, Hoffmann et al. studied a cohort of asthmatic patients and showed that the number of CD4^+^ CD25^+ ^T_Regs _was similar in allergic patients and control subjects [29]. Additional studies reported that the numbers of T_Regs _increased during the exacerbation of asthma [30, 31]. These findings are not consistent with the current results. These differences might be related to the varying ages and disease statuses of the patients in the different studies. In addition, several reports have suggested that the suppressive activity of CD4^+^CD25^+^ T_Regs _is affected by various factors including the type of allergen, allergen exposure, and individual allergic status [31]. Some studies also suggested that high levels of CD25 and CD25^hi^ T_Regs _in patients might represent the generation of induced or adaptive T_Regs _during the exacerbation of allergic inflammation as a consequence of the immune response[32] In the current study, we observed a decrease in the number of CD4^+^CD25^+^ T_Regs _during serious AR. Compared with control subjects, the total number of CD4^+^CD25^+ ^T_Regs _and CD4^+^CD25^hi^ cells was reduced in AR patients. We hypothesize, that because the numbers of CD4^+^CD25^+ ^T_Regs _was decreased during the exacerbation of AR, the suppression of Th2 responses by regulatory T lymphocytes was impaired, which subsequently leads to allergic type 2 immunity in the nasal mucosa and airways. 

In conclusion, AR patients with airway allergies had decreased numbers of CD4^+^CD25^+^ and CD4^+^CD25^hi ^T_Regs_, and the number of Treg cells was correlated negatively with total serum IgE levels. This suggests that lower numbers of regulatory T cells might be associated with inflammation of AR. 

## Acknowledgments 

This study was supported in part by research grant 12ZR1409500 from Natural Science Foundation of Shanghai City, and in part by A Foundation no. 20100470654 for the doctoral program of Higher Education Research Fund. 

## Conflict of interest 

The authors report no conflicts of interest. The authors alone are responsible for the content and writing of this manuscript. 

## References

**Table 1. Table1:** Clinical characteristics of the study participants.

Characteristics	AR (n = 20)	Controls (n = 16)
Alter (> Jahre)	31.2 ± 10.3	29.8 ± 8.5
Gender		
Male	70.0% (14/20)	62.5% (10/16)
Female	30.0% (6/20)	37.5% (6/16)
Symptom score (> 6)	12.3 ± 3.1	0
Serum total IgE (IU/mL)	312.8 ± 165.8	70.1 ± 14.6
